# Bio-Based Adhesives Formulated from Tannic Acid, Chitosan, and Shellac for Packaging Materials

**DOI:** 10.3390/polym15051302

**Published:** 2023-03-04

**Authors:** Urška Vrabič-Brodnjak

**Affiliations:** Department of Textiles, Graphic Arts and Design, Faculty of Natural Sciences and Engineering, University of Ljubljana, 1000 Ljubljana, Slovenia; urska.vrabic@ntf.uni-lj.si; Tel.: +386-(1)-200-32-82

**Keywords:** adhesives, chitosan, tannic acid, shellac, Japanese Knotweed, Canadian Goldenrod, paper, packaging, material analysis

## Abstract

The aim of this study was to develop bio-based adhesives that can be used for various packaging papers. In addition to commercial paper samples, papers produced from harmful plant species in Europe, such as Japanese Knotweed and Canadian Goldenrod, were used. In this research, methods were developed to produce bio-based adhesive solutions in combinations of tannic acid, chitosan, and shellac. The results showed that the viscosity and adhesive strength of the adhesives were best in solutions with added tannic acid and shellac. The tensile strength with adhesives of tannic acid and chitosan was 30% better than with commercial adhesives and 23% for combinations of shellac and chitosan. For paper from Japanese Knotweed and Canadian Goldenrod, the most durable adhesive was pure shellac. Because the surface morphology of the invasive plant papers was more open and had numerous pores compared to the commercial papers, the adhesives penetrated the paper structure and filled the voids. There was less adhesive on the surface and the commercial papers achieved better adhesive properties. As expected, the bio-based adhesives also showed an increase in peel strength and exhibited favorable thermal stability. In summary, these physical properties support the use of bio-based adhesives use in different packaging applications.

## 1. Introduction

Global economy depends on fossil resources, which provide raw materials for the production of a range of chemicals and materials for the manufacture of commercial products such as paper and packaging. As there is growing environmental awareness and the need to reduce the dependence on petroleum-based products, attention has been paid to the possibilities of synthesizing polymeric materials from bio-based renewable resources [1]. The paper and packaging segment dominates the market and is expected to grow further during the forecast period due to the robust growth in demand for packaging materials from food and beverage manufacturers and e-retail companies [2]. According to the global report, the sustainable packaging market size was estimated at USD 218.18 billion in 2020 and is projected to reach USD 441.41 billion by 2028, growing at a compound annual growth rate (CAGR) of 6.80% [3]. The eco-friendly packaging market is growing due to increasing environmental concerns arising from packaging waste ending up in landfills and polluting the environment. From 2009 to 2021, paper and cardboard were the main packaging waste in the EU (32.2 million tons in 2020), followed by plastic and glass [3]. The global production volume of paper and paperboard was approximately 417.3 million metric tons in 2021 [3].

In the paper industry, biomass, such as wood and other species, is undergoing constant change due to countries’ efforts to decarbonize, the rise of bio-based materials, and so on. The recent shortage of paper for various media, due to the shift from fiber to packaging applications, opens the space for alternative solutions. Recently, interest in the use of agricultural residues has increased. Invasive alien plant species are harmful to the environment on a larger scale where they occur. According to the European Union definition, invasive alien plant species are species that have been displaced from their natural ecological range by human activities and species whose introduction and spread outside their natural ecological range pose a real threat to biodiversity and the economy [4,5]. The impact of invasive plants has been studied and solutions for their removal and reuse have been presented by many researchers [6,7,8,9,10,11,12,13]. Such invasive plants in Europe are *Acacia*, *Fallopia Japonica* (known also as Japanese knotweed), Goldenrod, and many more, as listed in the list of Invasive Alien Species of Union concern [14].

Environmentally friendly packaging, so-called green packaging, based on biodegradable, recyclable, or compostable materials, is currently attracting a great deal of attention in many disciplines because of its unique properties compared to traditional petrochemical-based plastics. Green packaging materials play an important role in preserving and protecting the product. To fulfil these benefits, the bio-based material for green packaging should be made from materials that enhance the biopolymer properties of the packaging material and meet the requirements of the global market. In addition, to ensure the recyclability or biodegradability of the above packaging, bio-based adhesives should be included in the packaging process. 

Currently, the adhesive production is still based on by-products of petroleum processing, and with increasing concerns about environmental threats and sustainable development, the use of biodegradable and sustainable biomass to produce adhesives and other adhesives important to the industry is not only inevitable but also a response to reducing the impact caused by formaldehyde adhesives. The global adhesives market grew from USD 77.15 billion in 2022 to a projected USD 83.99 billion in 2023 at a compound annual growth rate (CAGR) of 8.9% [15]. There are several ways in which renewable materials can be incorporated into adhesives. The adhesives used in paper and cardboard packages are adhesives that are applied at high temperatures or are dispersion adhesives that are applied as water-based dispersions. Water-based adhesives are usually emulsions of thermoplastic resins because their properties result from the polymer used and the system used to emulsify the polymer in water. As with solvent-based adhesives, the water carrier is evaporated by air or diffused into the porous structure. After drying, the resulting adhesive can be a brittle, hard resin or a flexible film, depending on the adhesive formulation. The most commonly used emulsion-based adhesive is the polyvinyl acetate-polyvinyl alcohol copolymer, which hardens to a relatively rigid solid when water diffuses through the substrate, such as packaging material. Water-based adhesives are considered as substitutes for solvent-based adhesives for the purpose of reducing volatile organic emissions in various packaging processes (sealing the package with goods, etc.). Starch-based adhesives are the most used bio-based polymer in the paper industry. It has been proven that adhesives made from starch are not very stable when cooled during the production; moreover, the storage times of these adhesives are very short. Even with various modifications of starch, the limitation in terms of the moisture barrier remains. Although the use of soy proteins and dextrin offers advantageous properties for the development of bioadhesives, they have disadvantages in terms of water resistance, stability, and strength of the sheets, which are also the major drawbacks of the bio-based adhesives currently available on the market [16,17].

Therefore, bio-based adhesives could be an important complement to dispersion and solvent-based adhesives due to increasing environmental and health requirements [15,16,17,18]. Innovative dry, wet, and hot melt adhesives for packaging have been described in the scientific literature [18,19]. However, these are mostly based on synthetic polymers with a small addition of bio-based components. Among them are tannins, promising materials that could be obtained from the plants. Tannin adhesives are of increasing interest, especially in the wood, automotive, cord, and many other industries [20]. In particular, low resistance to moisture has led researchers to develop tannin-based adhesives blended or co-polymerized with resins and other additives. Tannin and other biopolymers such as chitosan and starch have also been used as packaging adhesives, as analyzed by Kaczmarek et al. and Marino et al. [20,21]. The combination of tannins with other polymers has shown that tannins can be considered as additives for bio-based adhesives due to their potential effects ranging from adhesive properties to important water resistance. Mentioned adhesives also play an important role as underwater adhesives, which are an important multifunctional bonding solution for biological and engineering adhesive applications [22]. Chitosan is known as a non-toxic biopolymer, derived from the deacetylation of chitin. Due to its high crystallinity, hydrogen bonds between molecular chains, which exhibit great oxygen properties, has also attracted a lot of interest in the packaging field and as an adhesive [23,24,25,26,27,28]. A natural polymer that is already used in many fields, as well as in packaging, is shellac. It is a natural polymer, obtained from purified resinous secretions by the insect Kerria Lacca (Kerr) Lindinger (Coccideae). This species is the most important lac insect, being a main source of lac, for the production of shellac. Shellac’s chemical structure is composed of hard and soft resins of polyesters and single esters containing hydroxyl and carboxyl groups [29,30,31]. It is widely used as adhesives, thermoplastics, insulating materials, sealants, and coatings in pharmaceutical and agronomical industries [32]. Shellac has excellent film-forming and barrier properties.

The aim of this research was to develop bio-based adhesives that could be used for paper-based and environmentally friendly papers (produced from European invasive plant species). It is known that coated papers or papers made from invasive plant species in Europe are mostly hydrophobic, with a dense fiber structure; therefore, finding a suitable adhesive is an important environmental issue. On the other hand, the aim was also to produce an adhesive that will be environmentally friendly, biodegradable, or compostable for different packaging papers. By combining different materials (tannic acid, chitosan, and shellac) with selected properties, various combinations of bio-based adhesives have been produced for different types of packaging papers. This study presents strategies for varying ingredients, solvent combinations, cure times, and temperatures, and how to produce strong adhesives when the natural components are degradable, nontoxic, and sustainable.

## 2. Materials and Methods

First, various natural adhesive solutions were prepared. After the preparation of the adhesive solutions, the analysis and the efficiency of adhesion to different packaging materials were carried out. The procedures are presented in the following chapters.

### 2.1. Materials

#### 2.1.1. Materials for Bio-Based Adhesives

Tannic acid (TA) and chitosan (CH) were commercial compounds purchased from the Sigma-Aldrich company (Merck KGaA, Darmstadt, Germany). The deacetylation degree of chitosan was 78% and its molecular weight was 1.8 × 10^6^. The molecular weight of tannin acid was 107.2 g/mol.

Shellac, a refined product obtained from LAC, which is the resinous secretion of the female insect Kerria Lacca (Kerr) Lindinger (Coccideae), was supplied from A. F. Suter & Co Ltd. (Witham, UK). In this analysis, Shellac ASL 10 solution was used, which is the purified lac (by physical absorption) according to Regulation EU 231/2012 and its specification for E904 Shellac. 

Preparation of adhesives:

- Tannic acid
- Tannic acid was dissolved in 0.1 M acetic acid (Merck KGaA, Germany) at a concentration of 2% and room temperature.
- Chitosan

N-acetylated chitosan was prepared by a modified N-acetylation process according to Fan et al. [33]. Chitosan (2 g) was dissolved in 2% (*v*/*V*) aqueous acetic acid (60 mL). The solution was diluted with 80 mL of methanol and stirred for 10 min. After the stirring, the acetic anhydride (acetic anhydride/amino group (n/n) = 1:4) was added to the diluted solution and stirred at room temperature for 15 min. The mixture was precipitated by 0.5 M KOH/aqueous solution to neutralize the solution. Before mixing shellac and chitosan, the chitosan solution was neutralized as described above to pH 7.5. 

#### Shellac

The 4 g of shellac ASL 10 and 10% (*w*/*w*) of PEG 400 (Merck KgaA, Germany) were stirred at 400 rpm for 10 min. Namely, the PEG 400 was added as a plasticizer to the shellac.

For the comparison of bio-based to commercial adhesives, polyvinyl acetate (PVAc) adhesive was used. 

Adhesives were prepared in mixtures with different ratios as presented in Table 1.

#### 2.1.2. Packaging Paper Used for the Adhesive Testing

Packaging materials used in this research were:

1. Commercial, non-coated paper, with a specified grammage of 200 g/m^2^ and thickness of 0.3 mm *.
2. Commercial, two-sided coated paper, with a specified grammage of 200 g/m^2^ and thickness of 0.4 mm *.
3. Papers produced from European invasive plant species such as Japanese knotweed and Canadian Goldenrod (properties of each paper are presented in the following chapters and Table 2).

* The composition of the paper was 60–85% groundwood and 15–40% chemical pulp with a total pigment content of 20–30%.

#### Papers Produced from Invasive Plant Species (IAPS)

Two different types of paper samples from invasive plant species in Europe were used in this study, i.e., paper from Japanese Knotweed (JK) and Canadian Goldenrod (CG). Papers from the JK and CG were produced in an Andritz paper machine (Andritz AG, Graz, Austria, located in Pulp and Paper Institute, Ljubljana, Slovenia). As the presented papers are not commercial yet, detailed fiber properties are presented in Table 2. Both papers were included in the pulp, softwood, hardwood, and alternative fibers (i.e., fibers from Japanese Knotweed and Canadian Goldenrod).

### 2.2. Methods

#### 2.2.1. Grammage, Thickness, Density, and Specific Surface Volume of Paper Samples

It was necessary to determine basic paper properties, which have an influence on other properties and are presented in the next chapters. 

Grammage was determined in accordance with the ISO 536 standard, where 10 samples of each paper were cut into sizes of 10 × 10 cm and weighed. The thickness of the paper samples was measured with a precision micrometer Mitutoyo (Mitutoyo Corp., Kawasaki, Japan) to the nearest 0.0001 µm at 10 random locations at each paper. From the grammage and thickness, the density and specific volume of each sample paper were calculated, as described in the ISO 534 standard.

#### 2.2.2. Viscosity, Drying, and Application of Adhesives to the Paper Samples

The viscosity of the adhesives was tested according to standard EN 12092:2002, using a rotational viscosimeter Brookfield (Anton Paar GmbH, Graz, Austria). The measurements were made with a spindle of stainless steel at a rotation speed of 1.5 rpm. 

Drying time is highly dependent on temperature. It was chosen to couple drying time and temperature. 

#### 2.2.3. Paper Smoothness and Porosity 

As paper consists of a randomly matted layer of fibers, the structure has varying degrees of porosity. To determine the adhesive efficiency, it is important to analyze the surface of the paper. The measurements were made on the smoothness and porosity instrument Bendtsen (Alat UJI, Indonesia). The measuring range was from 0 to 5000 mL/L and the air pressure was passed through a flat metal ring and the specimen. The pressure difference was read from the selected rotameter tube. Smoothness and porosity were measured on both sides. For all samples, 10 replicas were conducted.

#### 2.2.4. Tensile Tests

The tensile strength (TS) and elongation at break (E) of the unbonded and bonded paper specimens (butt joint and lap joint) were measured using an Instron testing machine (Instron Inc., Norwood, MA, USA) at the cross speed of 50 mm/min according to the ASTM D638-92 standard. The measurements were analyzed using the Instron Bluehill^®^ program. 

The amount of adhesives was different for each analysis, but the same for all samples:

1. Butt joint: edges of two butt joints, the same paper samples were immersed 2 mm into the adhesive solution and their wetted edges were then joined. All adhesives for butt joints were dried at 30 °C; for shellac, 55 °C for 180 min.
2. Lap joint: 50 mg of each adhesive solution was spread over the length of 12 cm. Each strip was joined with 700 mN of compression force for 10 s, at 30 °C; for shellac, 55 °C.

When analyzing the efficiency of an adhesive, the lap joint plays an important role for the packaging materials. The paper was first cut and then overlapped to a length of 12 mm using a specific adhesive.

#### 2.2.5. Peel Strength

The peel strength of the adhesives was quantified as described in the ASTM D1876-08 standard. The test was performed with a 22 N load cell, and the test strips were 2.5 cm wide and 30.5 cm long. The adhesives were applied to the same paper samples, dried as described in the lap joint tensile test, for each adhesive. Samples were tested at an angle of 180° with the crosshead speed of 0.3 mm/min. 

#### 2.2.6. Thermogravimetric Analysis (TGA)

The mass loss as a function of temperature of each adhesive and paper was quantified via thermogravimetric analysis with analyzer TGA Q5000 (TA Instruments Inc., New Castle, DE, USA). An amount of 10 mg of adhesive and separate paper samples were put into the alumina crucible. For tannic acid, chitosan, and shellac, the weight loss vs. temperature is known from the literature; therefore, the procedure was not performed. The method was dynamic TGA, meaning that the temperature continued to increase over time as mass was recorded. The temperature measuring range was between 10 and 600 °C and the heating range was 10 °C/min under 30 mL/min of nitrogen gas flow.

#### 2.2.7. SEM Analysis 

The surface morphologies of adhesive films on packaging materials were observed using scanning electron microscope JEOL JSM-6060 LV (Jeol, Tokyo, Japan). Surfaces were taken under magnifications of 300× and 500× at a 10 kV voltage. 

#### 2.2.8. Statistics

Results were analyzed using one-way analysis of variance (ANOVA) with a 95% confidence interval. For each analysis, a different number of specimens were tested, as described in each method used.

## 3. Results and Discussion

### 3.1. Basic Properties

Basic properties such as grammage, thickness, density, and specific volume were determined for all sample papers (Table 3). Grammage and thickness influenced mostly mechanical properties of the paper such as tensile strength, elongation at break, stiffness, water barriers, and optical properties of the paper. Differences in thickness were correlated to fiber length and non-uniform thickness. All papers were produced on the paper machines; therefore, there was no floc formation during the paper-making process, which could result in a higher thickness variation among each sample. The results of basic properties of commercial papers showed similar results: at the invasive plant papers, the Canadian Goldenrod was a bit thicker, with a dense fiber structure and higher grammage, compared to Japanese Knotweed. 

### 3.2. Viscosity and Drying Properties of Adhesives

Viscosity is strongly correlated with the adhesive properties of glue. The adhesive surface tension must be less than or equal to the surface energy of the material to achieve good molecular interaction. Drying time and temperature are important properties when the adhesion proceeds. The drying temperature was determined at the same temperature of 30 °C for almost all tests, except for shellac, which was dried at 55 °C. Namely, the shellac had a semi-crystalline structure, with the glass transition temperature in the range of 40–50 °C. Above this temperature, shellac was converted into soft flowable and thermoplastic material [34]. Therefore, the drying temperature was higher. The drying time was different at the lap joint for all samples (Table 4). The lap joint drying time was defined separately for each specimen and adhesive solution. 

The penetration of chitosan solutions into porous, adherent materials was determined from previous researchers such as Patel et al. and Mati-Baouche et al. [35,36]. The roughness of the adherent surface was of secondary importance compared to the porosity. The low surface tension of chitosan solutions and the viscosity allow good penetration into rough surfaces, which was the case at our paper surface. The results showed that the chitosan solution had a viscosity of 100.9 Pas. The addition of tannic acid decreased the viscosity between 80.8 and 84.7 Pas. According to the experimental results, the interaction of chitosan and tannic acid, bridging flocculation, electrostatic patch, and hydrogen bonding played an important role in flocculation as described by An et al. [37]. Namely, hydrogen bonds can be formed between the groups—OH and -NH_2_ of chitosan and—OH of tannic acid and it is not known which functional group (-OH or NH_2_) of the chitosan molecule. The situation is the same with the interaction types that are responsible for flocculation and which hydrogen bond contributes most to the flocculation of tannic acid [37]. 

Shellac increased the viscosity, compared to chitosan, and it was (90.9−97.1 Pas), as the hydrogen and ester bonds in chitosan and shellac promote the network structure of interchain entanglement between polymers [38]. A large number of molecular chains were formed by temporary cross-linking, which produced gelation characteristics of the prepared adhesive solution [38]. The results showed that the addition of tannic acid to the adhesive solution can effectively increase the viscosity and bond strength of the adhesive. In summary, the results showed that grafting tannin acid onto the chitosan and shellac can effectively change the adhesion of the adhesive to paper, as it was also determined at the following tensile analysis. 

### 3.3. Paper Smoothness and Porosity

**Figure 1:**
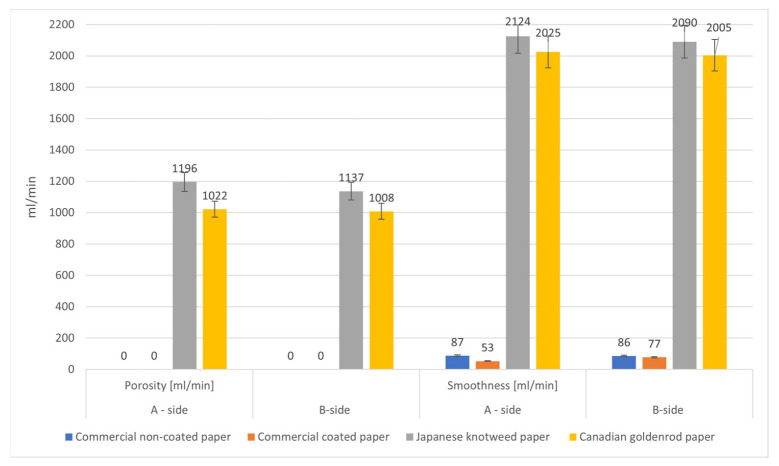
Smoothness and porosity of all paper samples, without adhesive.

Paper porosity is the ability of liquids and gases to penetrate the structure of paper and is a property of great importance in the use of paper. Paper is a very porous material; therefore, the porosity presents a critical factor in packaging materials and adhesive applications. Porosity is the measure of the total interconnected air voids, both vertical and horizontal, present in a sheet. The Bendtsen smoothness determines the amount of air that passes through the paper in one minute. In other words, it is an indirect measurement of roughness. That is, the higher the air pressure, the lower the resistance to air flow and the rougher the paper. Results of porosity and smoothness are presented in Figure 1. 

In the case of porosity, results were significantly different for commercial papers and papers from invasive plant species. Namely, the values of porosity were 0 at both commercial samples and between 1008 and 1196 mL/min for invasive plant papers.

In the current study, commercial papers were found to have a smoother surface, and papers from Japanese knotweed and Canadian goldenrod had a rougher surface, as expected. The A and B sides of the papers were almost the same, but on one side—the commercial-coated paper—the smoothness was better (53 mL/min).

### 3.4. Tensile Analysis

In the processing of paper packaging, where sealing is mandatory, are different types of closing and sealing, such as stapling, mechanical closing, and sealing with adhesive tape. Paper is considered as an anisotropic material as the fibers are aligned in the direction of the papermaking machine (i.e., machine direction—MD) and across the machine direction (CD). Tests were performed in both directions. Fracture of the adhesive joint may occur in several different ways such as cohesive failure of the adhesive, interfacial failure between the adhesive and paper surfaces, or cohesive failure of the paper/specimen. Tensile strength and elongation at break are very important for packaging materials and adhesive joints because of the special handling and shipping of the products. As the papers made from different materials, also from invasive plant species, could also be used as packaging materials, the properties were analyzed in detail. 

**Figure 2:**
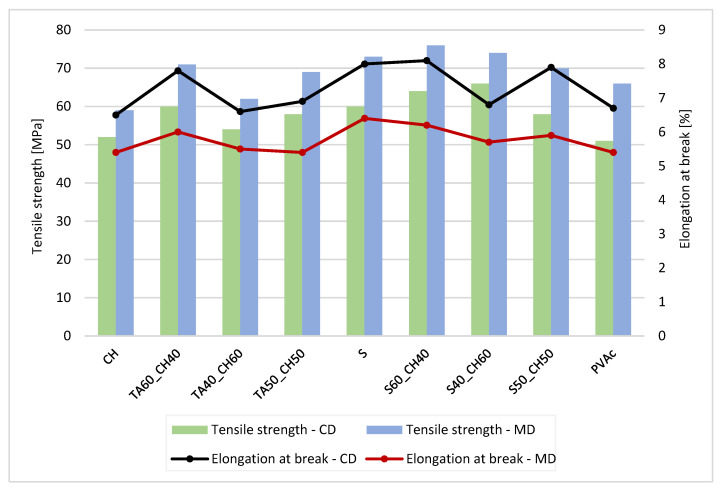
Tensile strength and elongation at break for commercial, non-coated paper with adhesive butt joints.

**Figure 3:**
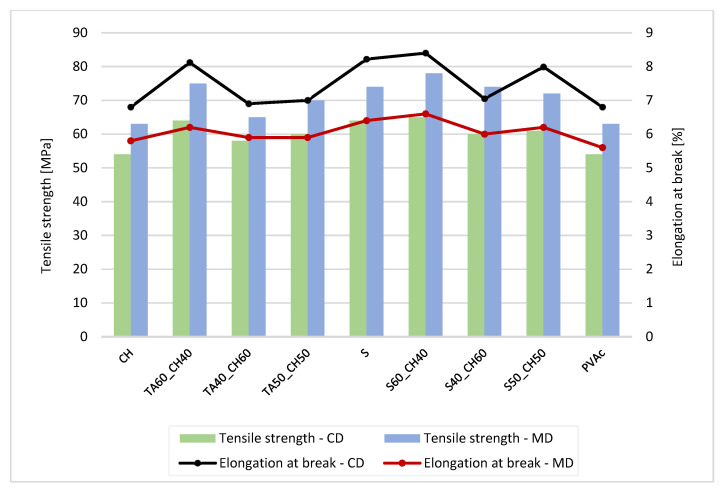
Tensile strength and elongation at break for commercial, coated paper with adhesive butt joints.

**Figure 4:**
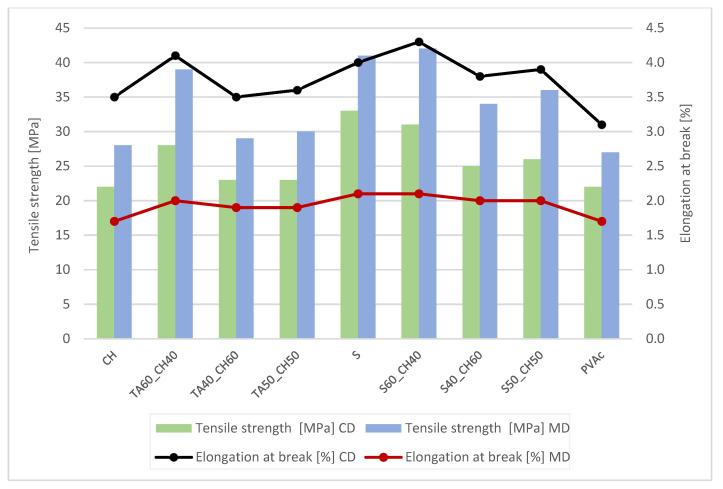
Tensile strength and elongation at break for Japanese knotweed paper with adhesive butt joints.

**Figure 5:**
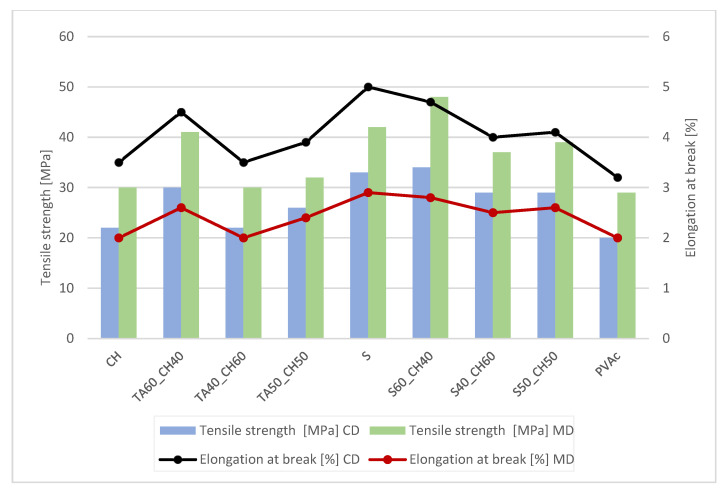
Tensile strength and elongation at break for Canadian goldenrod paper with adhesive butt joints.

Tensile strength (TS) and elongation at break (E) for each paper and type of lap joint adhesion are presented in Table 5, Table 6, Table 7 and Table 8. For butt joints, results are presented in Figure 2, Figure 3, Figure 4 and Figure 5. 

From Table 5, Table 6, Table 7 and Table 8, the results show that the highest lap joints had adhesives in the mixture with tannic acid (40%) and shellac (60%). The combination of tannic acid and chitosan gave also promising values of TS (63.11 Mpa in CD and 71.55 in MD direction) and E (8.19% in CD and 7.03% in MD direction). This indicates that the combination of tannic acid with chitosan and shellac controls the structural changes, and the matrix becomes more flexible, at invasive plant papers. With the higher amount of tannic acid, the solution increased the cohesive forces and fracture strength. When the amount of tannic acid decreased, the tensile properties deteriorated. Nevertheless, no significant difference in TS and E was noted between papers from Japanese Knotweed and Canadian Goldenrod. The values of Canadian Goldenrod were slightly lower, but no major differences between tensile strength and elongation at break were noted. 

In commercial, non-coated and coated papers, the tensile strength and elongation at break were higher than in papers from invasive plants. This was due to the coating, which had a less porous fiber structure in coated papers compared to papers from invasive plants. The tensile strength toward MD increased and the elongation decreased for all compounds and specimens, which was due to the interface of the adhesives with the molecular interactions between the fibers. The contribution of mechanical bonding was achieved by the mechanical anchoring of the adhesive in the pores of the paper surface and the embedding of the fibers protruding from the surface.

With the higher amount of tannic acid in the adhesive solutions, it was confirmed that it lowered the cohesion forces and the paper and joints (lap or butt) to break (Figure 2, Figure 3, Figure 4 and Figure 5). When the amount of chitosan was greater, the tensile strength improved for approx. 20 Mpa, but the elongation at break was lower, only approx. 3%. 

Compared to the sample papers without adhesive joints, the analysis showed that the papers with lap joint and bio-based adhesives were compact and stiff enough to hold the joint. At the same time and compared to the commercial adhesives, the bio-based adhesive blends exhibited promising adhesive properties.

Figure 2 and Figure 3 show that the tensile strength decreased with the addition of a smaller amount of tannic acid when the paper was butt-glued. The tensile strength was lowest when the amount of tannic acid in the adhesive mixture was 40%. The breaking point of the paper was in the glued joint (butt joint). For the lap joints, the breakage occurred in the paper itself, not in the glue joint. As expected, the butt joints were less rigid, yet the same trend as for the lap joint was observed, namely, that the tannic acid improved the adhesive properties of blends with other bio-based polymers.

For paper from Japanese Knotweed and Canadian Goldenrod, the most durable adhesive was pure shellac. When adding to the adhesive solution chitosan, the adhesive became more flexible (Figure 4 and Figure 5). A higher amount of tannic acid increased at both papers the tensile strength and it achieved better tensile results compared to the commercial PVAc adhesive. Chitosan as an adhesive, when dried, is resistant to water and temperature and possesses good mechanical properties, which was confirmed with this analysis. The addition of plasticizers to the adhesive solution increased the elongation and elasticity of the polymers and gave them greater resistance to mechanical stresses.

### 3.5. Peel Strength

The peel resistance of papers is an important property that is determined in terms of the needed force to peel at a defined rate and peel angle. The peel resistance depends on the adhesion of the adhesive to the surface, viscoelastic behavior of the adhesive and support material, and the temperature at which the procedure is performed. The results revealed no significant difference between the commercial samples. The difference was observed between the commercial and invasive plant papers, which was improved with the higher amount of tannic acid. 

**Figure 6:**
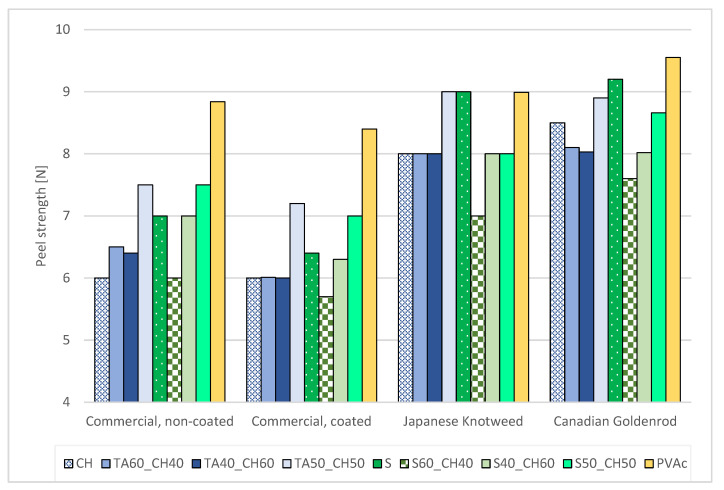
Average peel strength (N) for different adhesive formulations and paper samples.

The ANOVA results revealed differences among the paper samples, as shown in Figure 6. Especially the formulation with shellac and chitosan, at the commercial-coated sample, the peel strength was lowest among the tested samples. Namely, the adhesive was applied to the coated surface and the peel strength was 32% lower at the shellac and chitosan adhesive solution, compared to the PVAc adhesive. The best results were obtained with the biobased formulation using tannic acid in combination with chitosan. Shellac and pure adhesive showed great strength at invasive plant papers, which could be in line with higher porosity and stronger cohesive forces between the adhesive and specimen. 

### 3.6. Thermogravimetric Analysis (TGA)

**Figure 7:**
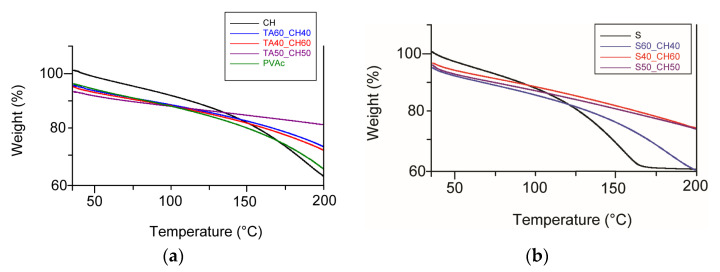
TGA analysis—weight loss versus temperature of adhesives such as (**a**) chitosan and combination chitosan with tannic acid and (**b**) shellac and combination chitosan with shellac.

Mass loss was measured separately by TGA for the adhesive combinations (Figure 7) to determine the amount of volatiles that could potentially migrate into the food if the materials and adhesives were used in this manner.

Crosslinked polymers such as tannic acid and chitosan showed two decomposition stages. The first occurred around 90 °C and the second one after 150 °C. This could be due to the high stability of the bond formed between the functional groups of chitosan and tannic acid, which requires a higher temperature to break the bond. In addition, the addition of TA resulted in an increase in the decomposition temperature compared to the control PVAc adhesive, indicating an improvement in the heat stability of bio-based adhesives.

Shellac is a semi-crystalline polymer that is regularly oriented and has a low density. It behaves like a polycrystalline material [39]. From the studies of Thombare et al., the crystallization of shellac decreases with decreasing temperature and melts at 90 °C [34]. The softening phase is between 65 and 70 °C. This process also depends on the cooling rates. The results of TGA analysis of shellac and the combination of chitosan showed changes in the pure shellac adhesive at temperatures higher than 150 °C. Decomposition occurred for the shellac first at 100 °C and then at 150 °C. When chitosan was added, decomposition still occurred, but less intensely and after 200 °C. As confirmed by many researchers, the low percent weight loss in the first decomposition phase from 20 °C to 100 °C may be due to evaporation of water and volatiles [35,36,37,38,39]. In contrast, the higher percentage weight loss in the second decomposition phase may be related to the decomposition of the biopolymeric materials, as shown in the results.

### 3.7. SEM Analysis

**Figure 8:**
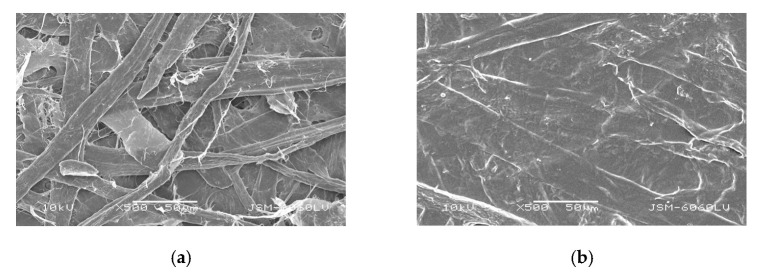
SEM images of surface (500× magnification) of commercial (**a**) non-coated and (**b**) coated papers.

To determine the relationship between the paper morphology and used adhesive solutions, samples were analyzed using a scanning electron microscope (SEM). Figure 8 shows surfaces of coated and non-coated paper. 

**Figure 9:**
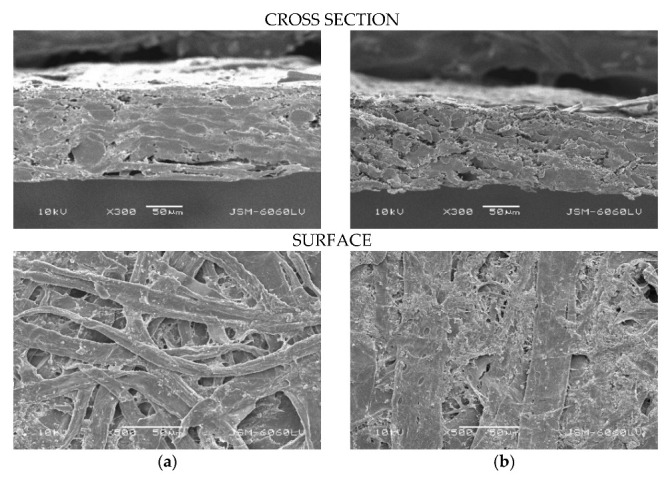
SEM images of cross-section (300× magnification) and surface (500× magnification) of papers from invasive plant species: (**a**) Japanese Knotweed (JK) and (**b**) Canadian Goldenrod (CG).

Figure 9 shows images of uncoated, unglued papers, clearly showing the surface and cross-section of the JK and CG papers. All micrographs of papers show the cross-section at 300× magnification and surface of 500× magnification. The paper from JK revealed an open, rougher surface compared to CG paper, which also confirmed lower values of tensile properties of CG. Namely, the surface of JK was more open and had more pores, which allowed the adhesive to penetrate the fiber structure. 

**Figure 10:**
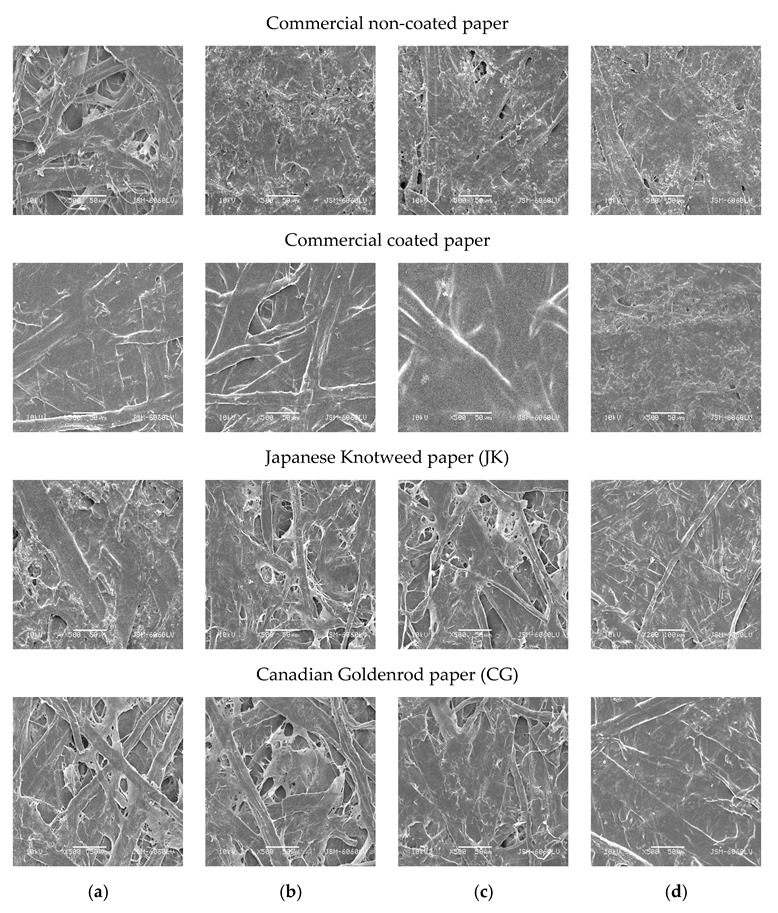
SEM images of papers from commercial non-coated and coated papers and invasive plant species Japanese Knotweed and Canadian Goldenrod with different adhesives applied: (**a**) 100% chitosan, (**b**) 50% tannic acid +50% chitosan; (**c**) 50% shellac +50% chitosan, (**d**) 100% PVAc.

The results of the surface and applied adhesive are shown in Figure 10 for all papers. These results were consistent with the changes in tensile properties and adhesive bonds. If the adhesive bond is uneven as a “coating,” then many properties also deteriorate. The adhesive solution penetrated the JK paper better than in the CG sample, as can be seen in Figure 10c, where the adhesive was more evenly distributed on the paper surface. The good penetration and distribution of the adhesive contributed to the observed increase in the impregnated (adhesive) part of the papers, especially the extensibility demonstrated in the tensile tests. The pore filling by the adhesive resulted in a matrix interspersed with the paper fibers, creating a pattern that is also used in reinforced fiber materials.

The experiments were run to verify the behavior of paper from invasive plant species bonded with bio-based adhesives as a promising recyclability solution. There are still studies and research to be conducted such as wettability, determination of recyclability, and potential industrial composting. 

### 3.8. Comparison between Fabricated Bio-Based and Commercial Packaging Adhesives Such as PVAc 

The combination of bio-based adhesives showed that they can be compared to commercial adhesives, such as the commonly used packaging adhesive PVAc, under similar conditions. When comparing the data, they indicate that less tannic acid was needed to formulate strong adhesive. From the previous research and our results, tannic acid might be considered as a multifunctional crosslinking agent [35,36,37]. Based on our results and other research, we conclude that the molecular interactions and structural properties of chitosan and shellac must contribute to the strong adhesion properties.

From the experiments presented in this research, it can be concluded that combination of tannic acid and chitosan and also chitosan and shellac can provide high-strength adhesive bonding. Tannic acid, as a plant polyphenol, can enhance the cohesive strength of the polymer network and, thus, impart better adhesive properties. The maximum adhesion for shellac and chitosan for invasive plant species papers was comparable to commercial glue. 

## 4. Conclusions

The bio-based adhesives produced and analyzed in this work are adhesives for dry substrates. In this research, tannic acid was confirmed as a suitable additive among the prepared adhesives. Indeed, the addition of tannic acid increased the viscosity and the adhesive strength (from 15 to 30%) and the properties were better compared to commercial adhesive. The tensile strength with adhesives of tannic acid and chitosan was 30% better compared to commercial adhesives and 23% better with combinations of shellac and chitosan. In the bottom joint of papers, the shellac adhesive combinations with chitosan showed the best properties. For paper from Japanese Knotweed and Canadian Goldenrod, the most durable adhesive was pure shellac. Microscopic analysis showed good filling of the paper voids by the adhesives. This is an important contribution to other factors for adhesion of cellulosic materials to other polymers. As the surface morphology of the papers from invasive plants was more open and had numerous pores compared to the commercial papers, the adhesives were able to penetrate the paper structure and fill the voids. As expected, the bio-based adhesives also exhibited a favorable thermal stability. In summary, these physical properties support the use of bio-based adhesives in different packaging applications.

To evaluate the feasibility of adhesive applications for food packaging and pharmaceutical products, further experiments and different substrates (such as films and foils) under different conditions and environments should be investigated in the future. The bio-based adhesives used show potential for the development of many more products and even more environmentally friendly technologies. Future analyses will focus on the biodegradability and/or recyclability of the adhesives investigated in this study. When using paper and bio-based adhesives, feasibility and commercial use in the future should answer questions about the environmentally friendly processing of the end products and their recyclability.

## Figures and Tables

**Table 1:** Weight ratios of each prepared adhesive mixture.

Adhesive Sample Name *	Adhesive Sample Combination	Weight Ratio
CH	Chitosan	100
TA60_CH40	Tannic acid + chitosan	60/40
TA40_CH60	Tannic acid + chitosan	40/60
TA50_CH50	Tannic acid + chitosan	50/50
S	Shellac	100
S60_CH40	Shellac + chitosan	60/40
S40_CH60	Shellac + chitosan	40/60
S50_CH50	Shellac + chitosan	50/50
PVAc	PVAc adhesive	100

* Chitosan—CH; Tannic acid—TA; Shellac—S; PVAc—polyvinyl acetate.

**Table 2:** Fiber properties of IPS used for paper production.

Properties	Japanese Knotweed Paper	Canadian Goldenrod Paper
Fiber length (mm)	0.775	0.452
Fiber width (µm)	18.66	13.85
Fiber orientation in paper sheets (°)	–30 to +30	–10 to +10
**Composition of the paper pulp**		
Softwood pulp (%)	27.5	27.5
Hardwood pulp (%)	27.5	27.5
Alternative fibers (%)	45.0	45.0

**Table 3:** Grammage, thickness, density, and specific volume of sample papers (presented are mean values with standard deviation).

Properties	Commercial, Non-Coated	Commercial, Coated	Japanese Knotweed	Canadian Goldenrod
Grammage (g/m^2^)	200 ± 2	199 ± 3	100 ± 3	105 ± 4
Thickness (µm)	0.298 ± 0.01	0.282 ± 0.04	0.123 ± 0.03	0.166 ± 0.02
Density (g/m^2^)	671 ± 2	521 ± 2	630 ± 1	550 ± 4
Specific volume (cm^3^/g)	1.490 ± 0.05	1.919 ± 0.07	1.590 ± 0.07	1.800 ± 0.04

**Table 4:** Properties of used adhesives.

Adhesive	Viscosity at 20 °C (Pa∙s)	Drying Time for Butt Joint (min)	Drying Time for Lap Joint (min)	Drying Temperature (°C)
CH	100.9	180	35	30
TA60_CH40	82.4	180	65	30
TA40_CH60	80.8	180	55	30
TA50_CH50	84.7	180	59	30
S	50.5	300	180	55
S60_CH40	90.9	180	90	30
S40_CH60	97.1	180	82	30
S50_CH50	95.0	180	86	30
PVAc	160	180	30	30

**Table 5:** Tensile strength and elongation at break for commercial, non-coated paper with adhesive lap joints.

Adhesive	Joints	Tensile Strength (MPa)		Elongation at Break (%)	
		CD	MD	CD	MD
Sample paper—no adhesive	Control—no joints	122.33 ± 0.15	134.11 ± 0.98	15.77 ± 0.99	13.52 ± 0.58
CH	Lap joint	85.24 ± 0.22	89.24 ± 0.82	9.95 ± 0.95	8.74 ± 0.63
TA60_CH40	Lap joint	109.08 ± 0.24	110.07 ± 0.99	9.74 ± 0.85	9.97 ± 0.88
TA40_CH60	Lap joint	100.07 ± 0.37	99.81 ± 0.14	12.05 ± 0.46	10.21 ± 0.92
TA50_CH50	Lap joint	90. 11 ± 0.14	95.44 ± 0.17	11.70 ± 0.51	11.43 ± 0.49
S	Lap joint	80.36 ± 0.84	94.21 ± 0.58	9.64 ± 0.67	9.08 ± 0.51
S60_CH40	Lap joint	81.82 ± 0.42	93.78 ± 0.22	9.08 ± 0.26	9.13 ± 0.27
S40_CH60	Lap joint	84.65 ± 0.55	95.43 ± 0.34	9.15 ± 0.82	8.81 ± 0.77
S50_CH50	Lap joint	83.12 ± 0.16	92.79 ± 0.67	10.22 ± 0.95	10.50 ± 0.96
PVAc	Lap joint	75.09 ± 0.07	89.05 ± 0.08	9.34 ± 0.13	8.08 ± 0.17

**Table 6:** Tensile strength and elongation at break for commercial, coated paper with adhesive lap joints.

Adhesive	Joints	Tensile Strength (MPa)		Elongation at Break (%)	
		CD	MD	CD	MD
Sample paper−no adhesive	Control—no joints	141.12 ± 0.01	157 ± 0.74	16.07 ± 0.96	14.82 ± 0.02
CH	Lap joint	84.51 ± 0.41	99 ± 0.52	10.14 ± 0.85	8.85 ± 0.14
TA60_CH40	Lap joint	88.62 ± 0.37	100 ± 1.08	11.23 ± 0.93	9.64 ± 0.43
TA40_CH60	Lap joint	110.73 ± 0.58	128 ± 0.23	13.45 ± 0.78	10.24 ± 0.90
TA50_CH50	Lap joint	99. 08 ± 0.72	109 ± 0.82	12.63 ± 0.96	11.22 ± 0.73
S	Lap joint	90.94 ± 0.16	104 ± 0.09	10.07 ± 0.53	9.85 ± 0.59
S60_CH40	Lap joint	90.55 ± 0.45	105 ± 0.14	9.98 ± 0.91	9.89 ± 0.76
S40_CH60	Lap joint	86.13 ± 0.82	95 ± 0.74	9.51 ± 0.77	8.62 ± 0.54
S50_CH50	Lap joint	93.74 ± 0.78	94 ± 0.52	11.17 ± 0.68	10.04 ± 0.99
PVAc	Lap joint	84.62 ± 0.84	96 ± 0.14	9.96 ± 0.81	8.53 ± 0.04

**Table 7:** Tensile strength and elongation at break for Japanese knotweed paper with adhesive lap joints.

Adhesive	Joints	Tensile Strength (MPa)		Elongation at Break (%)	
		CD	MD	CD	MD
Sample paper−no adhesive	Control—no joints	35.81 ± 0.38	37.52 ± 1.41	4.30 ± 0.28	2.00 ± 0.08
CH	Lap joint	38.22 ± 0.15	52.67 ± 1.02	7.85 ± 0.19	4.66 ± 0.34
TA60_CH40	Lap joint	63.11 ± 0.16	71.55 ± 1.41	8.19 ± 0.02	7.03 ± 0.02
TA40_CH60	Lap joint	49.23 ± 0.21	60.13 ± 0.35	7.05 ± 0.18	5.92 ± 0.84
TA50_CH50	Lap joint	53.08 ± 0.10	64.82 ± 0.17	7.27 ± 0.24	6.08 ± 0.02
S	Lap joint	72.5 5± 0.81	81.37 ± 0.42	12.67 ± 0.11	11.65 ± 0.27
S60_CH40	Lap joint	70.18 ± 1.09	79.60 ± 0.34	9.55 ± 0.08	7.16 ± 0.07
S40_CH60	Lap joint	56.39 ± 0.64	66.01 ± 1.58	7.93 ± 0.14	6.22 ± 0.39
S50_CH50	Lap joint	62.14 ± 1.20	70.24 ± 1.36	8.04 ± 0.19	6.56 ± 0.16
PVAc	Lap joint	35.01 ± 1.57	46.47 ± 0.99	7.00 ± 1.03	5.13 ± 0.21

**Table 8:** Tensile strength and elongation at break for Canadian goldenrod paper with adhesives lap joints.

Adhesive	Joints	Tensile Strength (Mpa)		Elongation at Break (%)	
		CD	MD	CD	MD
Sample paper—no adhesive	Control—no joints	39.45 ± 0.65	47.27 ± 0.09	2.75 ± 0.61	1.39 ± 0.13
CH	Lap joint	40.15 ± 0.18	53.67 ± 0.55	5.66 ± 1.05	3.92 ± 0.27
TA60_CH40	Lap joint	65.21 ± 0.22	80.34 ± 1.08	7.08 ± 0.98	5.39 ± 0.69
TA40_CH60	Lap joint	51.23 ± 0.07	68.92 ± 0.74	6.00 ± 0.23	4.30 ± 0.11
TA50_CH50	Lap joint	54.08 ± 0.35	70.04 ± 0.25	6.75 ± 0.56	4.54 ± 0.25
S	Control—no joints	73.19 ± 0.01	83.58 ± 1.92	8.69 ± 0.64	6.82 ± 0.09
S60_CH40	Lap joint	72.18 ± 0.75	82.60 ± 0.37	7.16 ± 0.81	5.72 ± 0.63
S40_CH60	Lap joint	57.39 ± 0.14	72.74 ± 0.92	6.94 ± 0.74	4.96 ± 0.41
S50_CH50	Lap joint	64.14 ± 0.89	77.14 ± 0.99	7.03 ± 0.11	5.02 ± 0.78
PVAc	Lap joint	38.01 ± 1.03	52.15 ± 1.54	4.91 ± 0.16	2.84 ± 0.52

## Data Availability

Not applicable.
